# Delayed Efficacy of Radiofrequency Catheter Ablation in Intramural Ventricular Arrhythmias: Implications for Timing of Repeat Ablation and ICD Implantation

**DOI:** 10.1002/ccr3.72713

**Published:** 2026-05-15

**Authors:** Chukwuka Elendu, Nwachukwu O. Nwachukwu, Courage O. Idahor, Chimaobi Nwevo, Nwamaka Chidera Bob‐Ume, Ebuka C. Ezeh

**Affiliations:** ^1^ Federal University Teaching Hospital Owerri Nigeria; ^2^ Darlington Memorial Hospital Darlington UK; ^3^ Henry Ford Health Detroit USA; ^4^ NYC Health + Hospitals/South Brooklyn Health Brooklyn USA; ^5^ All Saints University College of Medicine Kingstown Saint Vincent and the Grenadines; ^6^ Enugu State University College of Medicine Enugu Nigeria

**Keywords:** arrhythmia‐induced cardiomyopathy, delayed ablation efficacy, ICD timing, intramural ventricular arrhythmias, radiofrequency catheter ablation

## Abstract

Delayed efficacy of radiofrequency catheter ablation may occur in intramural ventricular arrhythmias due to progressive lesion maturation rather than immediate procedural failure. Recognizing this phenomenon may prevent premature repeat ablation or implantable cardioverter‐defibrillator (ICD) implantation. Careful post‐procedural observation and reassessment are essential to optimize clinical decision‐making and improve patient outcomes.

Dear Editor,

We read with great interest the recently published case report titled “Delayed efficacy of radiofrequency catheter ablation on arrhythmias originating in the interventricular basal septum” [1], which describes a patient with arrhythmia‐induced cardiomyopathy secondary to intramural idiopathic ventricular arrhythmias who demonstrated delayed suppression of premature ventricular complexes and subsequent recovery of left ventricular function following an initially unsuccessful radiofrequency catheter ablation (RFCA). The authors present a compelling and clinically relevant illustration of a phenomenon that remains underrecognized in routine electrophysiological practice—the delayed therapeutic effect of RF energy delivery in deeply located arrhythmogenic substrates. The clinical scenario described offers an opportunity to revisit several important mechanistic and clinical considerations related to intramural ventricular arrhythmias, procedural decision‐making after apparent ablation failure, and optimal timing for escalation of therapy such as repeat ablation or ICD implantation.

The central observation of delayed arrhythmia suppression following RFCA highlights the unique pathophysiology of intramural ventricular arrhythmias, particularly those originating from the basal interventricular septum. Unlike endocardial arrhythmogenic foci, intramural substrates are embedded within myocardial tissue and may be electrically insulated from both endocardial and epicardial surfaces. As described in the case, mapping identified early activation sites on both sides of the septum with only transient suppression during ablation, strongly suggesting an intramural origin [1]. This aligns with existing electrophysiologic understanding that similar activation timing across opposing septal surfaces, combined with the absence of a matching pace map, indicates a deep focus that may be inaccessible to conventional ablation strategies [2]. Such anatomical complexity creates inherent limitations in lesion depth and energy penetration, which may account for the delayed response observed.

From a pathophysiologic perspective, delayed efficacy following RFCA likely reflects the dynamic evolution of tissue injury rather than immediate electrical elimination of the arrhythmogenic substrate. Radiofrequency energy induces coagulative necrosis surrounded by zones of reversible injury and inflammation, with progressive lesion maturation occurring over hours to days [1, 3]. Subsequent inflammatory expansion, microvascular compromise, and fibrotic remodeling can enlarge the effective lesion volume beyond the initial ablation zone, eventually interrupting conduction pathways or eliminating surviving myocardial fibers capable of sustaining arrhythmia. This mechanism provides a plausible explanation for why arrhythmias may transiently persist or recur immediately after ablation yet resolve during follow‐up without additional intervention.

The case also underscores an important clinical challenge: distinguishing between procedural failure and incomplete lesion maturation. In contemporary practice, acute recurrence of ventricular arrhythmia following ablation often prompts consideration of repeat procedures or escalation to advanced strategies, including simultaneous unipolar ablation, bipolar ablation, infusion‐needle catheters, or epicardial access. While such techniques may improve lesion depth, they also carry increased procedural risk, particularly in septal regions adjacent to the conduction system or coronary vasculature. Recognition that delayed efficacy may occur—even after apparent procedural failure—suggests that immediate re‐intervention may not always be necessary and that a structured observation period could reduce unnecessary procedural exposure (Table 1).

**TABLE 1:** Practical considerations after apparent failure of radiofrequency catheter ablation for intramural ventricular arrhythmias.

Clinical context	Immediate interpretation	Suggested interim approach	Rationale
Transient suppression during energy delivery with early post‐procedural recurrence	Possible deep intramural focus with incomplete acute lesion expression	Short‐term observation with rhythm monitoring before repeat ablation	Delayed lesion consolidation may alter arrhythmia burden
Persistent high premature ventricular complex burden with stable hemodynamics	Potential incomplete substrate modification	Optimize medical therapy and reassess ventricular function before re‐intervention	Functional recovery may lag behind arrhythmia reduction
Reduced left ventricular ejection fraction with suspected arrhythmia‐induced cardiomyopathy	Reversible ventricular dysfunction	Serial imaging over weeks to months prior to ICD consideration	Structural recovery may occur after arrhythmia control
No acute noninducibility but anatomical evidence of deep septal origin	Substrate location limits immediate success	Consider advanced mapping strategies if recurrence persists after observation	Intramural substrates may require tailored procedural planning
Early recurrence in absence of symptoms or instability	Possible lesion maturation phase	Avoid immediate escalation unless clinical deterioration occurs	Prevents unnecessary procedural risk

Beyond procedural strategy, the implications of delayed ablation efficacy extend to device therapy decision‐making. In patients presenting with arrhythmia‐induced cardiomyopathy (AIC), the distinction between primary myocardial disease and reversible tachycardia‐mediated dysfunction is critical. As highlighted in the case, suppression of frequent premature ventricular complexes was followed by rapid improvement in left ventricular ejection fraction, supporting the diagnosis of AIC rather than irreversible cardiomyopathy [1]. Existing literature emphasizes that recovery of ventricular function after arrhythmia suppression may take weeks to months, and premature ICD implantation during this recovery phase may expose patients to unnecessary device‐related risks without long‐term benefit [4, 5]. The delayed response observed here reinforces the concept that waiting for electrophysiologic and structural remodeling after ablation may be prudent before committing to permanent device therapy.

Another noteworthy aspect of the reported case is the interplay between diagnostic uncertainty and procedural timing. Patients with frequent ventricular arrhythmias and reduced ejection fraction may present with overlapping features of primary cardiomyopathy, myocarditis, or arrhythmia‐induced dysfunction. In such settings, early procedural failure could be misinterpreted as evidence of a refractory substrate rather than a manifestation of lesion maturation dynamics. The case demonstrates how careful longitudinal reassessment—including serial imaging and rhythm monitoring—can clarify disease trajectory and guide management decisions more effectively than immediate procedural escalation.

Furthermore, the observation of delayed efficacy raises broader questions regarding endpoints for procedural success in ventricular arrhythmia ablation. Acute noninducibility and immediate suppression of ectopy are traditionally used as procedural markers; however, intramural substrates may challenge the reliability of these endpoints. Future procedural paradigms may benefit from incorporating markers of effective lesion delivery—such as transient suppression during energy application or characteristic impedance changes—as indicators of potential delayed success. Recognizing these signals could help operators balance procedural aggressiveness against safety considerations.

The mechanistic insights highlighted by these observations further emphasize the importance of understanding arrhythmia localization in guiding expectations for ablation outcomes. Ventricular arrhythmias arising from the left ventricular summit, basal septum, or deep myocardial regions have consistently been associated with lower acute success rates compared with superficial substrates [2, 6]. However, the possibility of delayed success suggests that acute procedural endpoints may underestimate long‐term efficacy in selected cases. Consequently, follow‐up strategies may require adaptation, including extended monitoring periods before declaring procedural failure.

Another critical implication relates to patient counseling. Individuals undergoing catheter ablation often expect immediate resolution of symptoms or arrhythmia burden. Awareness of delayed efficacy allows clinicians to set realistic expectations and prevent premature conclusions regarding treatment failure. Structured post‐procedural observation may reduce anxiety for both patients and clinicians while allowing time for lesion maturation.

From a research perspective, the phenomenon of delayed efficacy warrants further systematic investigation. Prospective studies evaluating predictors of delayed success—such as arrhythmia location, lesion parameters, or intra‐procedural electrophysiologic markers—could inform personalized management strategies. Additionally, integration of advanced imaging modalities such as cardiac magnetic resonance imaging or intracardiac echocardiography may help characterize lesion evolution and improve understanding of post‐ablation remodeling processes.

The authors' discussion of arrhythmia‐induced cardiomyopathy further underscores the reversibility of ventricular dysfunction once arrhythmia burden is reduced [1]. Pathophysiologic mechanisms including abnormal calcium handling, myocardial energy depletion, dyssynchrony, and extracellular matrix remodeling highlight the complex interplay between electrical and structural remodeling [4, 7]. Recognition that structural recovery can occur after delayed arrhythmia suppression reinforces the importance of cautious therapeutic escalation during the early post‐ablation period.

The mechanistic insights highlighted by these observations further emphasize the importance of understanding arrhythmia localization in guiding expectations for ablation outcomes. Similar delayed responses have been described following ablation of accessory pathways and supraventricular arrhythmias, suggesting that lesion maturation is a universal biological process rather than an anomaly [3]. Integrating this concept into clinical algorithms could improve patient outcomes by avoiding unnecessary interventions.

While the report provides valuable insight, it also highlights areas for further exploration. For example, standardized criteria defining when to observe versus re‐intervene after apparent ablation failure remain lacking. Development of consensus recommendations incorporating arrhythmia characteristics, lesion parameters, and patient risk profiles could assist clinicians in decision‐making. Additionally, advances in energy delivery techniques aimed at improving lesion depth while minimizing collateral damage may reduce reliance on delayed efficacy as a compensatory mechanism.

In conclusion, delayed efficacy following radio frequency catheter ablation may occur in patients with intramural ventricular arrhythmias and arrhythmia‐induced cardiomyopathy. Recognition that arrhythmia suppression may develop days after an apparently unsuccessful ablation, particularly when transient suppression is observed during energy delivery, has important implications for repeat ablation and ICD implantation decisions. Clinicians should consider an observation period following ablation of deep intramural substrates, particularly when transient suppression is achieved during energy delivery, as this may reflect effective lesion formation with subsequent delayed maturation. Increased awareness of this phenomenon may help optimize patient selection, reduce unnecessary procedural risks, and improve long‐term outcomes in complex ventricular arrhythmia management.

## Author Contributions

**Chukwuka Elendu:** conceptualization, investigation, data curation, methodology, formal analysis, writing – original draft, writing – review and editing, visualization, validation, project administration, supervision. **Nwachukwu O. Nwachukwu:** validation, writing – review and editing. **Courage O. Idahor:** validation, writing – review and editing. **Chimaobi Nwevo:** validation, writing – review and editing. **Nwamaka Chidera Bob‐Ume:** validation, writing – review and editing. **Ebuka C. Ezeh:** validation, visualization.

## Funding

The authors have nothing to report.

## Disclosure

The views expressed in this report are solely those of the authors and do not represent the official positions of any affiliated institutions. The authors declare no conflicts of interest and received no funding for this work.

## Ethics Statement

The authors have nothing to report.

## Consent

Written informed consent for publication was obtained by the authors of the original report. This letter does not include new patient data.

## Data Availability

Data sharing not applicable to this article as no datasets were generated or analysed during the current study.
